# Cardiac structure and systolic function in resistance-trained athletes versus untrained male adults

**DOI:** 10.1007/s00421-025-06060-1

**Published:** 2025-12-22

**Authors:** Abigail M. Saunders, Rebecca L. Jones, Joanna C. Richards

**Affiliations:** 1 Institute for Sport and Physical Activity Research, School of Sport Science and Physical Activity, University of Bedfordshire, Bedford, UK; 2 National Centre for Sport and Exercise Medicine, School of Sport, Exercise and Health Sciences, Loughborough University, Loughborough, UK; 3 Sport, Physical Activity, & Human Performance Research Group, School of Psychology, Sport Science and Wellbeing, University of Lincoln, Lincoln, UK; 4 Institute of Education, Arts and Community. Federation University, Victoria, Australia; 5 School of Sport, Exercise and Rehabilitation Sciences, College of Life and Environmental Sciences, University of Birmingham, Birmingham, UK

**Keywords:** Athlete’s heart, Hypertrophy, Echocardiography, Resistance exercise

## Abstract

**Purpose:**

Research examining the Athlete’s Heart has often focused on endurance athletes, yet no clear consensus has emerged on the cardiac adaptations observed in resistance-trained athletes. The purpose of this study is to examine cardiac structure and systolic function in resistance-trained athletes compared to untrained individuals.

**Methods:**

An observational cross-sectional study conducted echocardiographic examinations on male resistance-trained athletes (*n* = 12; body mass: 93 ± 19 kg [95%CI 82–104]) and age-matched untrained volunteers (*n* = 12; body mass: 80 ± 7 kg [95%CI 76–84]). Resting blood pressure and anthropometrics were gathered to allow indexing of cardiac parameters to body size and composition.

**Results:**

Compared to untrained individuals, resistance-trained athletes had greater septal (resistance-trained: 1.2 ± 0.1 cm; untrained: 0.9 ± 0.1 cm; *p* < 0.001), and posterior (resistance-trained: 1.2 ± 0.1 cm; untrained: 0.8 ± 0.1 cm; *p* < 0.001) wall thickness, and absolute left ventricular mass (resistance-trained: 275 ± 50 g; untrained: 162 ± 25 g; *p* < 0.01). These differences in left ventricular mass remained significant after accounting for body size and composition (*p* < 0.01). Cardiac dimensions of the resistance-trained athletes are greater than the normative range described in current literature. Significant differences in functional parameters including stroke volume, E/A ratio and LV end-systolic wall stress were also observed (*p* < 0.05).

**Conclusion:**

Both structural and functional differences in cardiac measures are apparent when comparing those engaged in chronic resistance-trained to untrained individuals. Furthermore, despite these structural differences, no significant impairment in left ventricular function was observed. However, whilst the cardiac dimensions of most resistance-trained athletes do not exceed the upper limits for physiological hypertrophy the exact mechanism for these differences is unclear and warrants further investigation.

## Introduction

Whilst the Athlete’s Heart has been extensively described in endurance-trained (ET) adults (Pluim et al. 2000; Utomi et al. 2013) disagreement remains among authors regarding this phenomenon in resistance-trained (RT) athletes (Utomi et al. 2013; Haykowsky et al. 2018). The Morganroth Hypothesis (Morganroth 1975) proposes that distinct patterns of cardiac remodelling arise in response to different modes of training and the associated haemodynamic demands. Specifically, the greater left ventricular mass (LVM) observed in chronically ET athletes was attributed to increased left ventricular internal diameter (LVID) and end-diastolic volume (EDV), reflecting a *volume overload*. In contrast, chronic resistance training, associated with acute elevations in arterial pressure and a *pressure overload*, was found to result in concentric hypertrophy, characterised by increased interventricular septum (IVS) and posterior wall (PW) thicknesses and LVM, with little or no change in chamber dimensions.

Subsequent research has found that RT athletes display some of the characteristics originally outlined in the Morganroth Hypothesis including greater IVS and PW thicknesses, and increased LVM when compared to untrained (UT) individuals (Utomi et al. 2013; Saunders et al. 2022). Furthermore, after indexing for body size and composition, LVM has been found to be greater in the RT athletes compared to the UT individuals (Caselli et al. 2011; Saunders et al. 2022) - contrary to previous research suggesting that the increase in LVM following chronic resistance training is merely reflective of the increased skeletal muscle and subsequent greater body size typically seen in these individuals (Naylor et al. 2008). The findings of these reviews suggest that RT individuals also display other characteristics of the Athlete’s Heart phenomenon (Utomi et al. 2013; Saunders et al. 2022). The studies reported greater LVID and EDV in the RT athletes compared with UT counterparts. These data consequently indicates that whilst left ventricular hypertrophy was apparent in the athletes, this may not be reflective of the concentric pattern first proposed by Morganroth (1975).

While most research in RT athletes has focused on cardiac structure and its relevance to the Morganroth Hypothesis, few studies have investigated cardiac function in this population. Saunders et al. (2022) reported that measures of systolic function, including left ventricular ejection fraction (EF), fractional shortening, stroke volume, cardiac output, and global longitudinal strain (GLS), did not differ significantly between RT athletes and UT individuals. This suggests that, despite structural adaptations in the left ventricle, resistance training does not appear to compromise systolic function, and aligns with the work suggesting that high intensity exercise training does not lead to adverse cardiac effects (Levine 2014). In terms of diastolic function, the same study observed marginally higher early mitral inflow velocity and E/A ratio in RT athletes (*p* < 0.05), while other diastolic parameters, including late mitral inflow velocity, deceleration time, and isovolumic relaxation time, were not significantly different. These findings suggest that some aspects of diastolic function, previously reported to be enhanced in ET athletes (Pluim et al. 2000; Utomi et al. 2013) may also be modestly improved in individuals undergoing chronic resistance training. Further studies directly comparing these populations are required to confirm this hypothesis.

The high study-to-study heterogeneity noted in these previous reviews (Utomi et al. 2013; Saunders et al. 2022) highlights the large variance between previous research and indicates the need for a more recent, reproducible study comparing these two populations using current echocardiographic techniques. With participation rates in resistance exercise increasing over recent years (Sports & Fitness Industry Association 2023), RT athletes of the 21st century may not reflect those examined in previous research and it is vital that a consensus regarding adaptations to functional, as well as structural, cardiac parameters is established.

The aim of this study was to use current echocardiographic techniques and indexing measures to examine differences in cardiac structure and systolic function in RT athletes compared to UT individuals. It was hypothesised that the RT individuals will display significantly greater LVM associated with wall thickening compared to their UT counterparts, in addition to maintained systolic and diastolic function.

## Methods

### Participants

An a priori power analysis based on an effect size reported by Caselli et al. (2011) (Cohen’s d = 1.2) indicated that 20 participants were needed to achieve > 80% power (α = 0.05; independent t-test), and 24 were recruited to account for a potential 20% attrition rate. Twenty-five healthy males initially volunteered to partake in the current study. One individual was later excluded after disclosing the use of anabolic steroids, leaving a final sample of 24 participants. These were evenly allocated into two groups: the RT group (*n* = 12; comprising four CrossFit athletes, four bodybuilders, two Olympic-style weightlifters, and two front-row rugby players) and the UT group (*n* = 12) - see Table 1. All individuals were free from any current or historical cardiovascular or peripheral disease and not on any prescribed medication known to impact cardiovascular mechanisms, for example beta blockers. All RT athletes trained a minimum of four times per week for > 2 years prior to the start of data collection and all competed regularly within their chosen discipline. The UT participants were not engaged in any current organised training programme or competitive sport and engaged in < 150 min of moderate intensity recreational activity per week for > 12 months prior to data collection. The habitual physical activity levels of participants were considered, and any participants involved in a large daily component of resistance exercise during their work (e.g., construction worker) were excluded.

**Table 1:** Participant demographic data

Parameter	Resistance-trained athletes (*n* = 12)	Untrained individuals (*n* = 12)	*p* value	Cohen’s d	Rank biserial correlation	
Age (years)	29 ± 4 [95%CI: 27–32]	30 ± 5 [95%CI: 27–33]	0.69	−0.16		
RT/week (hours)	7.3 ± 2.1 [95%CI: 6.2–8.5]	0.2 ± 0.3 [95%CI: 0.0–0.4]	**< 0.001**		1.00	
ET/week (hours)	0.8 ± 0.5 [95%CI: 0.5–1.1]	0.5 ± 0.6 [95%CI: 0.2–0.9]	0.23	0.50		
Duration (years)	6.3 ± 5.4 [95%CI: 3.3–9.3]	2.0 ± 0.7 [95%CI: 1.6–2.4]	**< 0.01**		0.69	
HT (cm)	181.7 ± 9.3 [95%CI 176.4–186.9]	182.2 ± 8.7 [95%CI: 177.3–187.2]	0.88	−0.06		
BM (kg)	93.0 ± 18.9 [95%CI: 82.3–103.7]	80.2 ± 7.3 [95%CI: 76.0–84.3]	0.06		0.46	
BSA (m^2^)	2.1 ± 0.2 [95%CI: 2.0–2.3]	2.0 ± 0.1 [95%CI: 2.0–2.1]	0.17	0.57		
FM (kg)	16.7 ± 8.1 [95%CI: 12.1–21.3]	16.3 ± 4.8 [95%CI: 13.5–19.0]	0.76		0.08	
MM (kg)	72.7 ± 11.3 [95%CI: 66.3–79.0]	60.8 ± 6.4 [95%CI: 57.1–64.4]	**< 0.01**	1.30		
BF (%)	17.2 ± 5.2 [95%CI: 14.2–20.2]	20.2 ± 5.3 [95%CI: 17.2–23.2]	0.18	−0.57		
RHR (bpm)	58 ± 9 [95%CI: 53–63]	65 ± 7 [95%CI: 61–70]	**0.04**	−0.88		
SBP (mmHg)	138 ± 11 [95%CI: 132–144]	135 ± 7 [95%CI: 131–138]	0.39	0.36		
DBP (mmHg)	82 ± 9 [95%CI: 77–87]	84 ± 9 [95%CI: 79–88]	0.64	−0.20		

*RT* Resistance training, *ET* Endurance training, *HT* Height, *BM * Body mass, *BSA* Body surface area, *FM* Fat mass, *MM* Muscle mass, *BF* Body fat, *RHR* Resting heart rate, *SBP* Systolic blood pressure, *DBP* Diastolic blood pressure. Presented as mean ± 1 standard deviation [95%CI (confidence intervals)]. **Bold** indicates significance (*p* ≤ 0.05). Effect sizes reported as small (*d =* 0.2–0.5), moderate (*d* = 0.5–0.8) and high (*d* ≥ 0.8)

Study procedures were approved by the institutional ethics review committee (ethical application: 2020ISPAR003) in accordance with the Declaration of Helsinki. Prior to data collection all participants provided informed consent. Members of the public were not involved in the design, reporting, or dissemination of the present study.

### Experimental protocol

An observational, age-matched control study was employed involving one familiarisation session and one main trial undertaken in the Sport and Exercise Science Laboratories at the University of Bedfordshire. Resting blood pressure (BP) was taken using an automatic BP machine (M3 HEM-7154-E, Omron, Kyoto, Japan), with height (cm) measured during full inspiration using a wall-mounted stadiometer (HAR-98.602, Harpenden, UK). A Tanita Body Composition Analyzer (MC-780MA, Tanita Corporation, Tokyo, Japan) was used to measure body mass (kg), body fat percentage (%), fat mass (kg) and muscle mass (kg) via bioelectrical impedance. Participants abstained from alcohol (24-hours), caffeine (12-hours), and strenuous exercise (48-hours) prior to data collection and were fasted for > 3 h as per manufacturer’s guidelines. Body Surface Area (BSA) was calculated as, $\:{\mathrm{0}}.{\mathrm{007184*}}\:({\mathrm{HT}}^{{{\mathrm{0}}.{\mathrm{725}}}} \:{\mathrm{*}}\:{\mathrm{BM}}^{{{\mathrm{0}}{\mathrm{.425}}}} )\:$where HT denotes height (m), and BM is body mass (kg).

An echocardiographic study was performed by a single researcher (AS) at rest using a commercially available ultrasound machine (Esaote MyLab Omega, Genova, Italy) and phased array transducer (PX 1–5) following the most recent guidelines from Mitchell et al. (2019). Additionally, an integrated three-lead electrocardiogram (ECG) recording was obtained using standard electrode placement. During the echocardiogram three consecutive cardiac cycles were recorded and stored on the system for each view to allow the averages for each measurement to be calculated. Left ventricular end-systolic wall stress (mmHg) was calculated using an equation adapted from Stöhr et al. (2017); LV σ = 0.334 *(TMP*((0.9*LVIDs)/((IVSs + PWs)/2)*(1+((IVSs + PWs)/2)/LVIDs)), where TMP denotes transmural pressure (mmHg), LVIDs is left ventricular internal diameter at end-systole (cm), IVSs is interventricular septal wall thickness at end-systole (cm) and PWs is posterior wall thickness at end-systole (cm). Image quality grading was conducted in line with the approach outlined by Beaumont et al. (2017).

### Statistical analysis

An a priori power calculation indicated that a minimum of 20 participants were required to detect power at > 80% (α = 0.05; independent t-test with 2 groups and 1 measurement) using G*Power (University of Düsseldorf, Düsseldorf, Germany (Faul et al. 2007)) and calculations from Caselli et al. (2011) examining absolute LVM (Cohen’s *d*: 1.2). A minimum of 24 participants were recruited for this study to account for a 20% drop-out rate. All statistical analyses were performed using Jamovi for Windows (Version 2.3, Sydney, Australia). Independent samples t-tests were used for the normally distributed data to analyse differences between the UT and RT individuals. Where Levene’s test suggested a violation in the assumption of equal variance, non-parametric Mann-Whitney U tests were used to analyse differences between the two groups. An effect size of 0.2–0.5 was defined as small, 0.5–0.8 as medium, and ≥ 0.8 as large (Cohen 2013). Statistical significance was set at *p* ≤ 0.05. Measurements are presented as mean ± 1 standard deviation (SD) [95% confidence interval [CI]]. All analyses were carried out in line with the checklist for statistical assessment of medical papers (CHAMP) statement (Mansournia et al. 2021).

### Equity, diversity and inclusion

The author group consists of different academic careers. All members of the research team are female from the same country and discipline, exercise physiology. Our study population is all male due to the impact of higher testosterone and lower sex hormone-binding globulin being found to be independently associated with a greater increase in LVM in males than females (Laurent et al. 2018). Participants were of mixed ethnic background. The generalisability of this data is considered in the discussion.

## Results

### Participant characteristics

No significant difference was found for age between RT (29 ± 4 years) and UT (30 ± 5 years; *t*(22)=−0.39, *p* = 0.69) individuals, see Table 1. As expected, training patterns differed markedly between groups, with the RT participants completing significantly more resistance exercise per week than UT individuals (RT: 7.3 ± 2.1 h [95% CI 6.2–8.5]; UT: 0.2 ± 0.3 h [95% CI 0.0–0.4]; U(22) = 0.0, *p* < 0.001). Both groups engaged in a small and comparable volume of endurance exercise per week (*t*(22) = 1.23, *p* = 0.23). The majority of RT participants (75%) competed at a sub-elite level, including regional and national competitions. The RT group had significantly lower resting heart rate than the UT group (*t*(22)=−2.17, *p* = 0.04); however, there were no significant differences between RT and UT for systolic (*t*(22) = 0.87, *p* = 0.39) or diastolic BP (*t*(22)=−0.48, *p* = 0.64).

### Body composition

No significant difference in body mass between the RT and UT group was evident (*U*(22) = 39.0, *p* = 0.06; Table 1). The RT and UT group were of similar height (*t*(22)=−0.15, *p* = 0.88) and BSA (*t*(22) = 1.40, *p* = 0.17) and fat mass (*U*(22) = 66.0, *p* = 0.76) see Table 1. The RT group presented with significantly greater muscle mass than the UT group (*t*(22) = 3.19, *p* < 0.01). Despite the significant difference in muscle mass and the lack of significant difference in body mass and fat mass, no significant difference in body fat percentage was found between RT and UT groups (*t*(22)=−1.40, *p* = 0.18).

### Cardiac structure

**Fig. 1:**
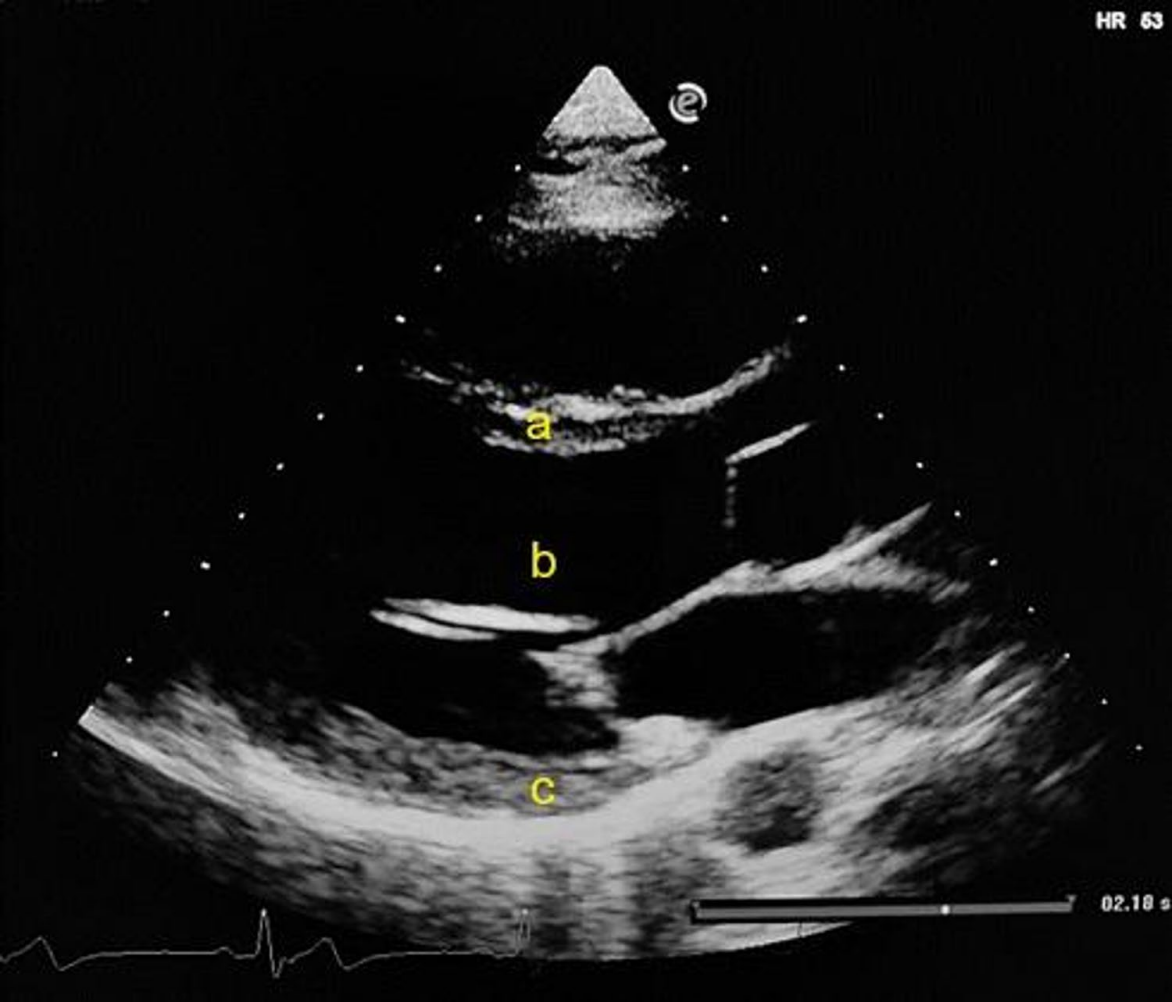
Sample echocardiographic image of a parasternal long-axis view acquired in the present study. Labels indicate key measurements: **a** interventricular septum (IVS), **b** left ventricular internal diameter (LVID), and **c** posterior wall (PW). All measurements were taken at end-diastole, defined the frame before mitral valve closure or the frame in the cardiac cycle in which the ventricular dimension or volume is largest, as recommended by Lang et al. (2015)

**Fig. 2:**
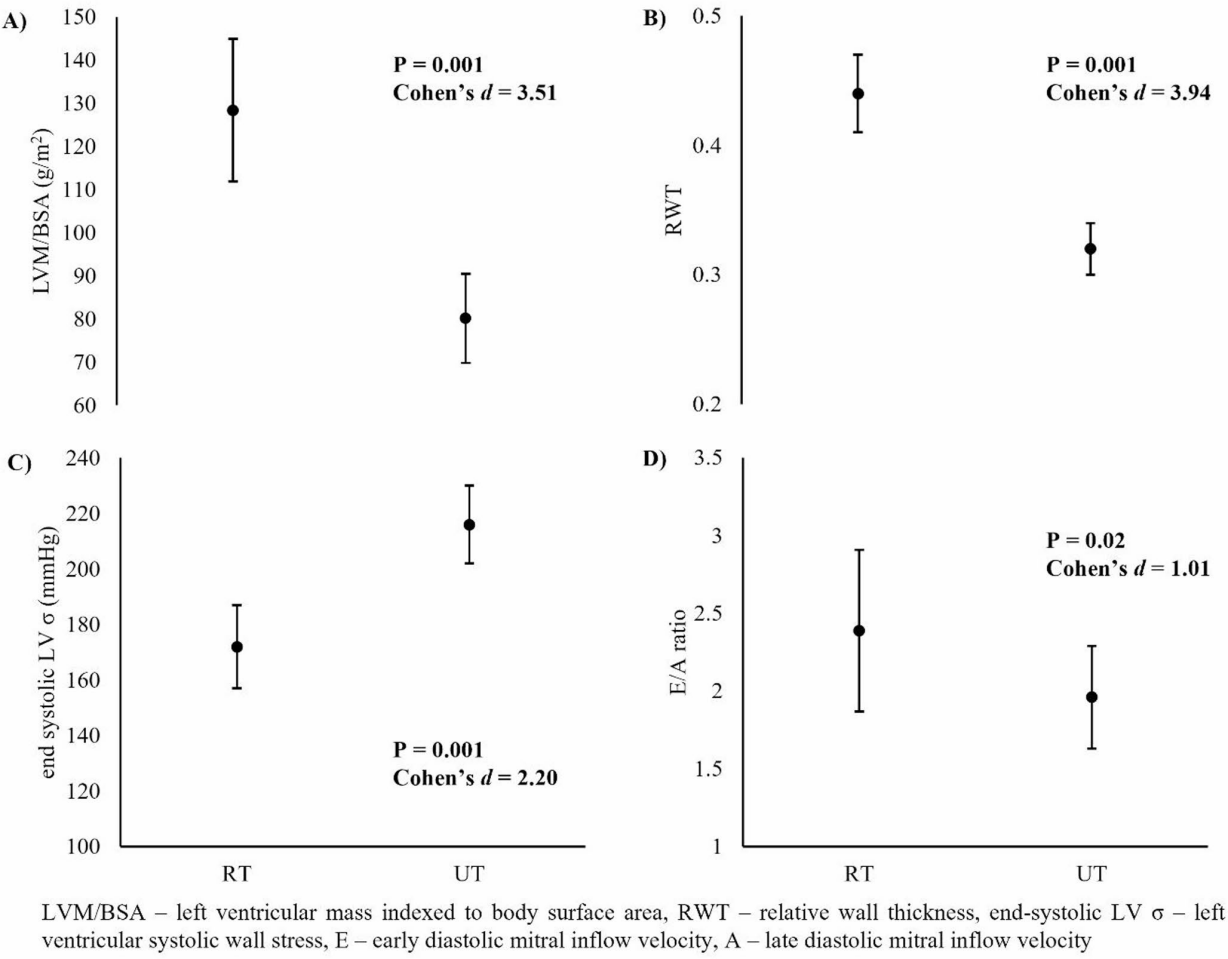
Cardiac outcomes measured using echocardiography, in resistance-trained and untrained male adults

Standard echocardiographic parameters for cardiac structure are presented in Table 2. Image quality was rated as good or excellent for 88% of echocardiographic images and a sample image of the parasternal long axis view is provided in Fig. 1. Echocardiographic reproducibility was high, with coefficients of variation (CoV) ranging from 0.9 to 7.2% for repeated measurements and 2.1–16.2% for image acquisition. The RT group had a significantly thicker IVS (*t*(22) = 9.5, *p* < 0.001) and PW (*t*(22) = 10.08, *p* < 0.001) than the UT group during diastole. This was also evident during systole for both IVS (*t*(22) = 9.46, *p* < 0.001) and PW (*t*(22) = 10.55, *p* < 0.001). There was no significant differences found between RT and UT groups for LVID during diastole (*t*(22) = 1.69, *p* = 0.11) or systole (*t*(22) = 0.20, *p* = 0.85). Relative wall thickness (RWT) was significantly greater in the RT athletes, compared to their UT counterparts (*t*(22) = 9.66, *p* < 0.001, Fig. 2).

**Table 2:** Echocardiographic left ventricular structure and left ventricular mass indexed to body dimensions

Parameter	Resistance-trained athletes (*n* = 12)	Untrained individuals (*n* = 12)	*p* value	Cohen’s d	Rank biserial correlation	
IVSd (cm)	1.19 ± 0.09 [95%CI 1.13–1.25]	0.86 ± 0.07 [95%CI 0.82–0.90]	**< 0.001**	3.87		
LVIDd (cm)	5.42 ± 0.36 [95%CI 5.22–5.62]	5.19 ± 0.31 [95%CI 5.02–5.36]	0.11	0.69		
PWd (cm)	1.16 ± 0.11 [95%CI 1.10–1.23]	0.81 ± 0.05 [95%CI 0.78–0.84]	**< 0.001**	4.12		
IVSs (cm)	1.29 ± 0.11 [95%CI 1.23–1.35]	0.95 ± 0.06 [95%CI 0.91–0.98]	**< 0.001**	3.86		
LVIDs (cm)	4.07 ± 0.52 [95%CI 3.77–4.36]	4.03 ± 0.27 [95%CI 3.88–4.19]	0.85	0.08		
PWs (cm)	1.31 ± 0.10 [95%CI 1.25–1.36]	0.92 ± 0.08 [95%CI 0.88–0.96]	**< 0.001**	4.31		
LA	3.87 ± 0.48 [95%CI 3.60–4.14]	3.25 ± 0.36 [95%CI 3.05–3.45]	**< 0.01**	1.47		
LVM (g)	275.19 ± 49.98 [95%CI 247–303]	161.63 ± 24.67 [95%CI 148–176]	**< 0.001**		0.99	
LVM/BM (g/kg)	2.99 ± 0.39 [95%CI 2.76–3.21]	2.03 ± 0.32 [95%CI 1.84–2.21	**< 0.001**	2.67		
LVM/FM (g/kg)	19.19 ± 7.04 [95%CI 15.20–23.17]	10.93 ± 4.05 [95%CI 8.64–13.22]	**< 0.01**		0.72	
LVM/MM (g/kg)	3.79 ± 0.49 [95%CI 3.51–4.07]	2.67 ± 0.40 [95%CI 2.44–2.90]	**< 0.001**	2.49		

*IVS* Interventricular septum, *PW* Posterior wall, *LVID* Left ventricular internal diameter, *d* Diastole, *s* Systole, *LA* Left atrial diameter, *LVM* Left ventricular mass, *LVM/BM* Left ventricular mass indexed to body mass, *LVM/FM* Left ventricular mass indexed to fat mass, *LVM/MM* Left ventricular mass indexed to muscle mass. Data presented as mean ± 1 standard deviation [95%CI (confidence intervals)]. **Bold** indicates significance (*p* ≤ 0.05). Effect sizes reported as small (*d =* 0.2–0.5), moderate (*d* = 0.5–0.8) and high (*d* ≥ 0.8)

Whilst no significant difference in LVID was found between the two groups, the RT athlete did present with a significantly greater left atrial diameter (*t*(22) = 3.61, *p* < 0.01) and larger left ventricular outflow tract (*t*(22) = 2.26, *p* = 0.03) diameter compared to the UT group. Absolute LVM was significantly greater in the RT group than the UT group (*U*(22) = 1.00, *p* < 0.001). When indexing to BSA, the RT athletes displayed significantly greater LVM/BSA (*t*(22) = 8.59, *p* < 0.001) compared to the UT group (Fig. 2). Significant differences were found when examining further indexing parameters including body mass, muscle mass, fat mass and body fat percentage and can be found alongside effect sizes in Table 2.

### Cardiac function

Standard echocardiographic parameters for cardiac function are presented in Table 3. The RT athletes had significantly greater EDV (*t*(22) = 4.80, *p* < 0.001) and end-systolic volume (*U*(22) = 16.00, *p* < 0.001) compared to UT individuals. There was no significant difference was found for EF between the RT and UT groups (*t*(22) = 2.02, *p* = 0.06). Furthermore, the RT group had a significantly greater left ventricular outflow tract velocity time integral (*t*(22) = 2.57, *p* = 0.02) and stroke volume (*t*(22) = 2.91, *p* < 0.01) compared with the UT group.

**Table 3:** Cardiac function measured using echocardiography, in resistance-trained and untrained male adults

Parameter	Resistance-trained athletes (*n* = 12)	Untrained individuals (*n* = 12)	*p* value	Cohen’s d	Rank biserial correlation
EDV (ml)	178 ± 30 [95%CI 161–195]	132 ± 14 [95%CI: 124–140]	**< 0.001**	1.96	
ESV (ml)	85 ± 15 [95%CI 76–94]	66 ± 6 [95%CI 63–69]	**< 0.001**		0.78
EF (%)	52.6 ± 2.5 [95%CI 51.2–54.0]	50.0 ± 3.8 [95%CI 47.8–52.1]	0.06	0.83	
SV (ml)	77.3 ± 27.3 [95%CI 61.9–92.6]	53.1 ± 9.2 [95%CI 47.9–58.3]	**< 0.01**	1.18	
E (cm/s)	0.70 ± 0.10 [95%CI 0.64–0.76]	0.62 ± 0.06 [95%CI 0.59–0.66]	**0.03**	0.93	
A (cm/s)	0.30 ± 0.04 [95%CI 0.28–0.32]	0.32 ± 0.04 [95%CI 0.30–0.35]	0.14	−0.63	
DT (ms)	181.6 ± 33.8 [95%CI 162.4–200.7]	150.5 ± 21.4 [95%CI 138.4–162.7]	**0.01**	1.10	
IVRT (ms)	81.4 ± 14.2 [95%CI 73.4–89.5]	88.6 ± 11.7 [95%CI 82.0–95.3.0.3]	0.19	−0.55	
Endocardial GLS (%)	−18.7 ± 2.5 [95%CI 17.3–20.1]	−18.4 ± 1.6 [95%CI 17.5–19.3]	0.42		0.20
Epicardial GLS (%)	−17.6 ± 2.4 [95%CI 16.2–19.0]	−16.9 ± 1.6 [95%CI 16.0–17.9]	0.43	0.33	

*EDV* End-diastolic volume, *ESV* End-systolic volume, *EF* Ejection fraction, *SV*- Stroke volume, *E* Early diastolic mitral inflow velocity, A late diastolic mitral inflow velocity, *DT* Deceleration time, *IVRT* Isovolumic relaxation time, *GLS* Global longitudinal strain. Data presented as mean ± 1 standard deviation [95%CI (confidence intervals)]. **Bold** indicates significance (*p* ≤ 0.05). Effect sizes reported as small (*d =* 0.2–0.5), moderate (*d* = 0.5–0.8) and high (*d* ≥ 0.8)

Whilst the RT group displayed a significantly greater E wave velocity (*t*(22) = 2.28, *p* = 0.03), no significant difference was found between the two groups for A wave velocity (*t*(22)=−1.53, *p* = 0.15). Consequently, E/A ratio was significantly greater in the RT individuals compared to the UT individuals (*t*(22) = 2.48, *p* = 0.02; Fig. 2). RT athletes displayed a significantly greater deceleration time (*t*(22) = 2.69, *p* = 0.01) compared to UT individuals. Isovolumic relaxation time was not significantly different between RT and UT groups (*t*(22)=−1.35, *p* = 0.19). At rest, end-systolic LV σ was significantly lower in the RT athletes compared to the UT individuals (t(22) = 5.38, *p* < 0.001; Fig. 2). There was no significant difference in GLS in the endocardial layer (*U*(22) = 57.50, *p* = 0.42) or in the epicardial layer (*t*(22) = 0.81, *p* = 0.43) between the RT athletes and UT counterparts.

## Discussion

The aim of this study was to investigate cardiac structure and systolic function of RT athletes compared to age-matched UT individuals at rest. The present study highlights that while RT athletes display structural adaptations, these changes do not appear to compromise cardiac function at rest. Following echocardiographic analysis, this study found that RT athletes displayed some key differences in cardiac dimensions when compared to the UT individuals, including a greater LVM associated with significant wall thickening. Furthermore, when compared to the most recent validated guidelines (Lang et al. 2015) these RT athletes displayed greater LVM (275.19 g versus 200.00 g), IVS (1.3 versus 1.0 cm) and PW (1.2 versus 1.0 cm) than the upper range of normative values. Whilst previous studies have found significant wall thickening in RT athletes (Utomi et al. 2013; Saunders et al. 2022), these individuals often present with significantly higher resting BP compared to UT individuals (Lattanzi et al. 1992). It has therefore been suggested that cardiac wall thickening occurs as a response to a constant pressure overload and mimics the response seen in individuals with pathological pressure overload conditions (Grossman et al. 1975).

Interestingly, in the present study, average resting BP did not significantly differ between the RT athletes and UT individuals. However, we acknowledge that according to current guidelines (Whelton et al. 2022), both groups would fall into the elevated/stage 1 hypertensive category, which could potentially influence cardiac structure and function. No participants were reported to be on anti-hypertensive therapy. As such, while the observed adaptations in the RT athletes are likely related to resistance training, the potential contribution of elevated BP cannot be fully excluded. As such, if the increased wall thicknesses witnessed in the RT athletes are a response to BP, then it is likely due to the acute significant elevations that have previously been described during heavy lifting (MacDougall et al. 1985), rather than due to an underlying constant pressure overload. While previous research has found a marked increases in arterial BP during resistance exercise (MacDougall et al. 1985; Haykowsky et al. 2001) reported the actual pressure to which the heart is exposed (the transmural pressure) is lower at intensities close to one-repetition max due to the involuntary implementation of a Valsalva manoeuvre. Future research should examine acute pressure responses to resistance exercise across various intensities – with and without the implementation of a Valsalva manoeuvre – to increase understanding of how this may impact cardiac adaptations within present day RT athletes.

Additionally, the current study highlights differences in LVM between RT athletes and UT individuals even after accounting for variations in body composition. When indexing to BSA, LVM was significantly greater in the RT athletes compared to UT individuals, and previous reported upper limits of normative data (115.00 g) (Lang et al. 2015), as was RWT (normal limits < 0.43). The greater relative LVM and RWT suggests the RT group present with concentric hypertrophy (Haykowsky et al. 2002). Although the current RT sample comprised athletes practicing different RT modalities- which Haykowsky et al. (2002) suggested may elicit differing LV geometry due to varying cardiovascular responses- no consistent differences in geometry were observed between subtypes. Training data in the present study were obtained via self-report from the athletes, including time spent in resistance versus endurance training. Future research could strengthen these findings by quantifying training loads using objective measures such as accelerometers to reduce potential self-report bias. The current study may therefore suggest that competitive RT athletes with > 2 yrs experience do exhibit a concentric pattern of LV hypertrophy. That said, whilst BSA is most frequently used as an indexing measure for echocardiographic data (Naylor et al. 2008), this parameter does not consider difference in body composition and recent work has found lean body mass to be a more appropriate – especially for RT athletes (Rato and Richards 2022). The current investigation found that LVM remained greater in the RT athletes compared to UT individuals when indexing to muscle mass, fat mass and body mass – demonstrating that a greater cardiac mass was present irrespective of the greater total body or skeletal muscle mass that was also observed. These data (Caselli et al. 2011; Saunders et al. 2022) are in contrast to the argument by Naylor et al.(2008) that the greater LVM in RT athletes is directly proportional to the participants’ greater body size and presence of skeletal hypertrophy. However, given that the significant difference in LVM between the two groups remained even after indexing to muscle mass, this suggests that the greater LVM of RT athletes is a true physiological adaptation to training rather than merely reflective of significant differences in body size.

While no significant difference was observed in LVIDd between the RT and UT participants, LVIDd is limited by its singular dimension and may fail to identify chamber dilation where chamber dilation is present (Gibson et al. 2014). The moderate effect size for LVIDd, in addition to the significantly greater EDV in the RT athletes compared to both the UT group and reported normative data (Lang et al. 2015), indicates that some degree of chamber dilation is present in RT athletes - suggesting that current resistance training may include elements which cause a different haemodynamic demand to that which Morganroth et al. (1975) initially proposed.

Despite the greater left ventricular volumes, no significant difference was found for EF between the RT and UT individuals, aligning with previous research (Caselli et al. 2011; Utomi et al. 2013), suggesting that despite significant changes to cardiac morphology there are no obvious impairments to systolic function following chronic resistance training. The use of GLS has been applauded for its reliability and increased prognostic value over EF (Lang et al. 2015). In the present study, whilst EF was lower than the normative range (52–72%) described by Lang et al. (2015), GLS for both RT and UT participants were like previously reported reference values (−19.1%) (Bussadori et al. 2009).That said, differences among vendors and software pacages are still vast, and universal normal values for GLS are still yet to be firmly established so recommendations should be taken with care (Lang et al. 2015).

The higher E wave and consequent greater E/A ratio, in the RT athletes compared to the UT individuals is not indicative of a normal transmitral flow pattern (Mottram 2005). However, when compared to normative values (Lang et al. 2015) neither a reduced A wave, abnormally fast deceleration time or shortened isovolumic relaxation time were noted in the RT group of this study. It is therefore likely that this ratio instead represents improved passive diastolic filling and a favourable suctioning effect from the left ventricle during early diastole (Armstrong and Ryan 2018), which could be explained by the significantly lower heart rate in this group. Furthermore, while this high ratio is not uncharacteristic of RT athletes (Mert et al. 2018), this directly contradicts previous meta-analyses (Utomi et al. 2013; Saunders et al. 2022). Data is limited in this athletic population, and it is possible the lack of significant difference in diastolic function previously observed between the RT and UT individuals may be confounded by undisclosed steroid use and high study-to-study heterogeneity between past studies (Saunders et al. 2022).

Body composition was evaluated using bioelectrical impedance, a technique previously validated for use in athletic and non-athletic populations (Campa et al. 2022); however, dual-energy X-ray absorptiometry is recommended as the ‘gold standard’ for body composition analysis (Shepherd et al. 2017), especially when examining athletes who are likely to have significant body composition differences when compared to healthy, but UT individuals of a similar age and ethnicity. Echocardiography was used to examine cardiac parameters, given its high accessibility and to align with previous research examining cardiac structure and function in athletic populations (Pluim et al. 2000; Utomi et al. 2013; Saunders et al. 2022). Magnetic resonance imaging has been described as the ‘gold standard’ for cardiac structural assessment (Utomi et al. 2013). However, when examining the electricity use, related consumables, and associated waste for diagnostic imaging, mean carbon-dioxide emissions for ultrasound scanning are substantially smaller than magnetic resonance imaging (McAlister et al. 2022), a key consideration for future researchers. Technological advances in 3D echocardiography are beginning to supersede the limitations of linear structural measurements, and future investigations of LV structure will likely be enhanced with 3D imaging.

## Conclusion

The present study suggests that RT athletes display wall thickening and consequently greater LVM than UT individuals. Consequently, RT athletes often present with structural cardiac dimensions greater than the upper limit of current normative data (Lang et al. 2015). However, in most individuals, these parameters do not exceed the upper normal limits for physiological hypertrophy (Pelliccia et al. 1993). Importantly, despite these structural adaptations, the RT athletes in this study did not demonstrate significant systolic or diastolic dysfunction. These findings provide insight into how the heart may adapt to the training stimulus typically experienced by resistance athletes. While these findings help establish expected cardiac parameters in the majority of RT athletes, individual variability exists. It should not be assumed that all RT athletes will display this pattern of cardiac remodelling or favourable functional adaptations. Furthermore, clinical evaluation is recommended - particularly if symptoms are reported - to rule out any underlying pathology.

## Data Availability

Data are available upon reasonable request. All data relevant to the study are included in the article or uploaded as supplementary information.

## Abbreviations

BP: Blood pressure
BSA: Body surface area
CI: Confidence interval
ECG: Electrocardiogram
EDV: End-diastolic volume
EF: Ejection fraction
ET: Endurance-trained
IVS: Interventricular septum
LV σ: Left ventricular end-systolic wall stress
LVID: Left ventricular internal diameter
LVM: Left ventricular mass
PW: Posterior wall
RT: Resistance-trained
UT: Untrained

## Declarations

### Patient involvement

No.
